# Parks and the Pandemic: A Scoping Review of Research on Green Infrastructure Use and Health Outcomes during COVID-19

**DOI:** 10.3390/ijerph182413096

**Published:** 2021-12-11

**Authors:** Megan Heckert, Amanda Bristowe

**Affiliations:** Department of Geography and Planning, West Chester University, West Chester, PA 19383, USA; AB967412@wcupa.edu

**Keywords:** green infrastructure, COVID-19, pandemic

## Abstract

Green infrastructure (GI) has long been known to impact human health, and many academics have used past research to argue for the potential importance of GI as a mechanism for maintaining or improving health within the context of the COVID-19 pandemic. This scoping review addresses the question: What evidence, if any, have researchers found of a relationship between green infrastructure use and health during the COVID-19 pandemic? Specifically, evaluating the (a) association of GI use with COVID-19 disease outcomes and (b) association of GI use with other health outcomes as impacted by the COVID-19 pandemic. Twenty-two studies were identified that measured GI use and studied it in relation to health outcomes during the pandemic. The studies were reviewed for the specific measures and types of GI use, level of analysis, specific types of health outcomes, and the conclusions reached with regard to GI use and health. Studies exploring COVID-19-specific health outcomes showed mixed results, while non-COVID health outcomes were more consistently improved through GI use, particularly with regard to improved mental health. While the evidence strongly suggests that GI use has played a protective role in non-COVID-19 physical and mental health during the pandemic, questions remain with regard to possible impacts on COVID transmission and mortality.

## 1. Introduction

Parks, gardens, and other forms of green infrastructure (GI) have long been demonstrated as having positive health benefits for surrounding communities. Early proponents of urban parks saw them as not only important spaces for ameliorating the negative environmental conditions associated with the industrial revolution, but also as key spaces for recreation and socialization [1]. Research would go on to demonstrate a wide range of health impacts of GI, including benefits for both physical and mental health. Physically, exposure to parks and other forms of vegetation has been associated with overall better health [2,3], with specific effects including reduced recovery times after surgery [4], reduced asthma [5], reduced obesity rates [6], and reduced incidence of chronic health conditions [7]. For mental health, natural areas are associated with reduced stress and anxiety [8], street trees were associated with lower rates of antidepressant prescriptions [9], and higher access to nature as children is associated with lower neuroticism in adults [10]. Even small green spaces such as greened alleys have been shown to have positive health effects [11].The mechanisms for these impacts on health are both direct and indirect. In some cases, the green infrastructure itself has direct health effects, such as when parks are used for exercise or when views of greenery improve moods and recovery times. Other mechanisms are less direct, such as lowered heat mortality due to reductions in the urban heat island effect brought about by trees and green spaces [12], or improvements in air quality as trees filter particulate matter and other pollutants from the air [13,14], positively affecting respiratory illnesses. The WHO has identified nine different pathways through which green spaces have been demonstrated to improve health, including direct pathways such as improved relaxation and less direct pathways such as improved social capital [15].

Though it is not uncommon for health benefits to be ascribed to GI generally, the reality is that there are many different forms of green infrastructure and not all of them will have the same kinds of impacts on human health. A greater normalized difference vegetation index (NDVI) value is associated with increased walking, lower body mass index (BMI) in children, lower rates of depression and anxiety, and lower mortality from cardiovascular and respiratory diseases. Higher green space density was also associated with lower mortality rates. Urban green infrastructure, such as street trees, green roofs, and other streetscape greenery, can also have a positive impact on health as residents with higher street tree density had lower rates of antidepressant prescriptions and street trees may also have positive impacts against stress. Proximity to green infrastructure has been shown to promote physical activity, as those who live closer to green infrastructure were more likely to meet government recommendations for the amount of physical activity. In addition, local green infrastructure can also have a protective effect on anxiety and depression [15].

In its review of the health impacts of urban green spaces, the World Health Organization highlighted that even within a single type of GI—urban parks—differences in size, amenities, and structure, as well as neighborhood context can affect the health impacts that result. High-quality green infrastructure is important for promoting physical activity and general health as the attractiveness of green infrastructure is associated with increased recreational walking. In addition, the quality of public open spaces such as parks and gardens has a greater impact on mental health than the quantity. However, large parks were associated with substantial increases in moderate to vigorous activity in children. Green infrastructure features such as paved trails and playgrounds are also associated with greater levels of physical activity. Greener residential areas and proximity to forests were associated with lower rates of childhood obesity. The presence of nearby trees and grass was shown to reduce mental fatigue in residents. A greater variability in the types of green infrastructure present can have a protective effect against coronary disease and stroke as residents have access to more aesthetically pleasing green spaces that can encourage walking [15]. Many of these benefits of green infrastructure suggest the potential for green infrastructure to play a role in the COVID-19 pandemic. Quite a few GI researchers have written commentary and other articles advocating for the potential importance of GI during the pandemic, given its known health benefits [16,17,18,19]. There are numerous potential mechanisms through which GI might influence health during the pandemic—potentially influencing both the progression of the pandemic and general health as we navigate lockdowns and protective measures. Might exposure to green infrastructure have a protective effect against COVID-19 as it has in the case of other respiratory ailments? Might it assist in recovery as it has been demonstrated to do after surgery? Given that COVID-19 outcomes are strongly influenced by underlying health conditions [20], might the positive relationship between GI and overall health translate to a positive association between GI and COVID-19 outcomes? If GI is indeed protective, could GI use serve as a form of primary prevention?

Additionally, because the pandemic has been quite different from other illnesses in its pervasiveness and impacts on general living conditions, more extensive impacts also seem possible. For example, might exposure to GI mitigate the potential negative mental and physical effects of isolation during lockdowns? Parks in particular have long been seen as important locations for physical activity and thus physical health—might that relationship become even more important during the pandemic when other locations such as gyms are closed or more difficult to access? Time spent is nature has been demonstrated to be restorative for both mental and physical health [21]; will that translate to pandemic conditions? Community gardens have been shown to increase social cohesion due to the facilitation of interactions among neighbors [22]; might GI serve as a safe space for interactions to battle pandemic-related isolation and its anticipated negative effects on mental health?

It must also be acknowledged, though, that the literature on green infrastructure and health is also not only positive. There is evidence of potential negative health outcomes related to use and exposure to GI, including potential exposure to allergens and chemicals produced by plants or used in their maintenance, exposure to disease, and risk of injury [15]. In the context of the COVID-19 pandemic, one must additionally ask if parks might serve as locations of transmission for the illness. While it is agreed that outdoor transmission of COVID-19 is rare, it is still possible and therefore does pose a risk [23]. In addition, certain forms of GI or outdoor activities may also encourage close contact, increasing the potential of contracting COVID-19. Yet, while outdoor transmission does pose some risk, practices can be implemented to make outdoor activities and GI use safer, such as masking and social distancing. Even if outdoor transmission of COVID is low, fear of outdoor transmission may still be important. While time in nature is generally restorative, that has not held true when the natural area evokes fear [24]. Might anxiety over potential COVID transmission prevent GI users from experiencing the mental health benefits of time in nature during the pandemic?

At the time of this writing, more than a year and a half into the pandemic, researchers are beginning to offer answers to some of these questions about the specific role of GI during the pandemic. For example, a recent review of built environment impacts on COVID found that the availability of parks was associated with lower risk of COVID transmission [25].

The term GI itself is used in many different ways in different contexts, sometimes referring broadly to a network of multiple forms of vegetation and green spaces, while used in other contexts to refer to green interventions or projects specifically designed to address particular environmental concerns, often stormwater management [26]. While this paper opts to focus on the more expansive view of GI, this means grappling with its many different forms and the possibility that different forms, existing in different contexts, impacted health in different ways and experienced different impacts. This review seeks to broadly address the question: What evidence, if any, have researchers found of a relationship between green infrastructure use and health in the context of the COVID-19 pandemic? Within this context, we sought to explore evidence of two types of health impacts: (1) impacts on the COVID-19 pandemic itself, and (2) impacts on non-COVID-19 health outcomes, particularly those that have been influenced by the pandemic.

We opted to focus specifically on GI use rather than presence to ensure comparability of studies. Our primary concern here was that restrictions on mobility varied so much both geographically and temporally that the presence of GI would be expected to differ considerably in its relationship to use based on local restrictions, such that in some cases GI proximity would be a proxy for use and exposure while in other cases, with more restrictive lockdowns, it might not. This focus on use necessarily restricts the GI types included in the study to forms of GI that can be “used” or “visited”, such as parks and gardens, and excludes general measures of vegetation and tree canopy.

## 2. Materials and Methods

As noted above, the term green infrastructure is used to mean different things in different contexts. Some use the term to refer to all natural or vegetated areas, while others use it to refer to a set of stormwater management practices, many of which use vegetation to reduce stormwater runoff [27]. For this review, we focused largely on the former definition. However, as noted above, our focus on use necessarily restricts that definition slightly to those forms of GI that can be said to be used. This includes a narrower range of green infrastructure forms such as parks, natural areas, and gardens.

**Figure 1:**
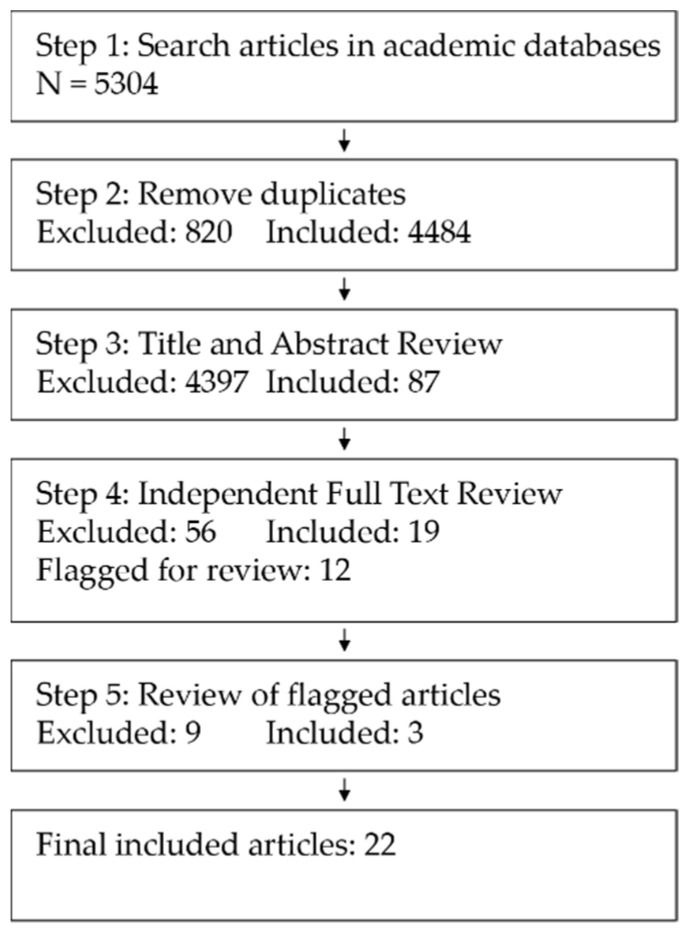
Diagram of the search process.

This study was designed following the JBI guidelines for the conduct of scoping reviews [28]. We identified articles using a five-step process. First, we conducted a systematic search of two academic databases: EBSCOhost’s Academic Search Ultra and Scopus. We conducted a series of searches between October 16 and 20, 2021, run for articles with titles, abstracts, or keywords including the term COVID-19 in addition to at least one of a list of GI-related terms. The GI search terms used were parks, greenspace, green space, green infrastructure, gardens, vegetation, nature, and natural lands. We restricted our search to peer-reviewed articles published in English. Article details were imported into Mendeley citation management software (Elsevier, Amsterdam, Netherlands), and duplicates were identified and removed. Article titles and abstracts were read to assess suitability for the review based on exploring potential links between GI and health outcomes during the COVID-19 pandemic. All remaining articles were then independently reviewed by both researchers reviewing the full text based on the more explicit criteria of: (1) reporting the findings of an empirical study, (2) measuring GI use, and (3) explicitly testing the relationship between GI use and health outcomes. The two reviewers compared results and identified areas of disagreement. These were discussed and included or excluded only with agreement of both reviewers. Figure 1 provides a diagram of the search process. In reviewing the articles, the works cited sections were further reviewed for additional articles to include, though no additional articles were added through this process.

Identified articles were then read closely and notes taken as to the measure of GI use, the level of analysis, the health outcomes measured, and the conclusions reached. Studies were assigned to the two themes of COVID-19-related and non-COVID-19 health outcomes. Results within them were reviewed further and discussed by the researchers to identify potential additional questions to consider. The COVID-19 outcomes articles were further charted with regard to locations, methods, and, when available, proposed mechanisms for GI use impacts.

## 3. Results

Ultimately, 22 articles were included in the full review. After detailed reading and charting on the articles, they were sorted into two themes: those reporting findings related to impacts of GI use on COVID health outcomes and those reporting findings related to impacts of GI use on non-COVID health outcomes. The full list of articles and theme designations is provided in Appendix A. The remainder of this section reports the details of articles included in each of the two themes.

### 3.1. Theme 1: Studies Exploring the Impact of GI Use on COVID-19-Specific Health Outcomes

Seven articles explored the potential impacts of green infrastructure use on COVID-19-related outcomes. These articles were reviewed with regard to the measure of GI use, unit of analysis, specific health outcomes, and findings with regard to GI use impacts. Details are provided in Table 1. Note that all seven studies use Google community mobility data on park use as their measure of GI use, so that section has been removed from the chart for this theme.

Six articles touched on a potential relationship between green infrastructure use and COVID-19 transmission as measured through case rates, while two assessed a relationship with COVID-19 deaths, and one measured COVID-19 reproductive rates. There were no systematic findings with regard to COVID-19 outcomes, with similar numbers of results indicating negative impacts, positive impacts, and no impacts of park use. Two of the articles found varying results within the study, as Kartal, Depren, and Depren [32] noted park use as related to higher COVID-19 deaths but having no impact on cases. Tyrovolas et al. [35], by contrast, found an overall pattern of park mobility indicating lower COVID-19 spread, except in areas of lockdown, where it was associated with higher spread. Of the four studies that found a positive association between park mobility and COVID-19 outcomes, two specifically noted that park results were weaker than other forms of mobility [30,33].

With the initial charting results showing such varied findings, we further reviewed each study with regard to locations, variables and relationships considered, and proposed mechanisms for the impact of GI use on COVID-19 and the geographic extent and scale of the study. Those more detailed results are provided in Appendix B. This closer look reveals a wide variation in locations included in this research, including one global study that also specifically addressed the regions of Africa, Latin America, and the Caribbean [35], and country-specific studies of England [31], India [34], Portugal [29], Turkey [32], and the US [30,33]. As previously noted, all studies used Google community mobility data that provided aggregated mobility scores for six sets of destinations: retail and recreation, grocery and pharmacy, parks, transit stations, workplaces, and residential destinations. The data were derived from cell phones and compared to baseline mobility metrics from January and February 2020. Detailed descriptions of the Google data were provided in all seven studies. Six of the studies used the mobility data directly, while Johnson et al. [31] transformed the data to measure park mobility as a proportion of overall mobility. Not all of the studies specifically addressed the park mobility metric with regard to mechanisms of impact, typically focusing instead on mobility in general as an indicator of the lack of social distancing. Those studies that discussed parks typically indicated the lower impact of parks in comparison to other forms of mobility and highlighted the potential lower risk of transmission in outdoor, open settings.

### 3.2. Studies Using GI as Explanatory Variables to Understand Other Health Outcomes within the Context of the Pandemic

We identified 15 articles that used a measure of GI use as an explanatory variable to understand non-COVID physical and mental health outcomes within the context of the pandemic and social distancing restrictions. These articles were reviewed with regard to the measure of GI use, unit of analysis, specific health outcomes, and findings with regard to GI use impacts. Details are provided in Table 2. Note that all fifteen studies use individuals as their unit of analysis, so that section has been removed from the chart for this theme.

Studies in this theme included both a wider array of outcomes and a wider range of GI variables than the first theme. Health outcomes included a range of mental health measures including anxiety, depression, and stress, physical health outcomes including sleep quality, somatization, and fatigue, and general outcomes including wellbeing and life satisfaction. GI variables included use of public parks and green spaces, use of private green spaces such as gardens, and unspecified time in “nature” which may have represented public or private property. Though the measures were more varied for these studies in comparison to the COVID-19-specific health studies, the outcomes were more consistent, as fourteen of the fifteen studies reported some positive impacts of green infrastructure use during the pandemic. Because health outcomes tended to be worse overall during lockdown compared to pre-pandemic measures and reports, the positive impact of GI was most often indicated not by improvements over pre-pandemic conditions but by less severe negative outcomes compared to those with lower GI exposure.

## 4. Discussion

Less than two years after the World Health Organization declared COVID-19 a global pandemic, a large literature on green infrastructure and its role during the COVID-19 pandemic has emerged, exploring the potential impacts of GI on health in the context of COVID-19. Though the general purpose of this scoping review is to describe the current state of the literature and catalog existing evidence, this section will discuss key findings and open questions from each of themes as well additional takeaways that emerge from this broad look at the literature.

### 4.1. Impacts of GI Use on COVID-19-Related Health Outcomes

While an initial look at the seven studies of GI use and COVID-19 outcomes suggests mixed results, a closer look reveals that the studies offer some level of agreement. In particular, they suggest a broad finding that while mobility in general is connected to worse COVID-19 outcomes, the type of mobility matters, and that park mobility is revealed as less risky than other forms of mobility even when positively associated with COVID-19 cases. This is particularly demonstrated by Johnson et al. [31], who specifically tested park mobility as a proportion of overall mobility rather than the raw data of park mobility. As the proportion of park mobility increased, COVID-19 case rates decreased. This relationship held across locations, but was particularly strong in areas with low park availability and more contiguous parks. The strong indication here is that park use helped to lower COVID-19 incidence insofar as it served to replace other forms of mobility that may have been riskier. While no other studies directly looked at park mobility as a replacement for other forms of mobility, two studies noted that while park mobility was associated with worse COVID-19 numbers, the association was lower for parks than for other mobility destinations [29,33], and two studies found no association between park mobility and COVID-19, while associations did exist for other mobility factors [30,34]. What emerges then is the strong suggestion that mobility in general increases risk, but park mobility is relatively safer and has lower negative outcomes than other forms of mobility. Risk of transmission outdoors is now generally perceived to be low, but specific pathways of outdoor transmission are not yet fully understood [23]. There is also a need for further study of demographic differences in COVID-19 outcomes with regard to GI.

The literature on GI use and COVID-19-specific health outcomes to date relies entirely on Google mobility data aggregated to coarse geographies. This represents a significant methodological limitation, as this approach cannot definitively speak to outcomes at the individual level. In particular, there is no clear proof that those people visiting parks are the same people who are or are not contracting or dying from COVID-19. Individual-level analysis in which the COVID-19 status of GI users and non-users was tracked would be particularly useful for understanding whether the value of GI use varies based on socio-demographic characteristics, a question which is also currently unstudied, as only one of the included studies included demographics of any kind, and that study only considered the proportion of the population over age 70 [31]. The exclusive focus on park use additionally represents a limitation as far as understanding the impacts of GI. While the Google “parks” category is broad, it is focused on public spaces. As the second category of papers highlighted, private green spaces such as yards and gardens might also be important, but were not considered in these studies. Individual-level analysis would additionally enable this more nuanced perspective on GI types.

More information is also needed on potential geographic variation in the impacts of GI use. The studies so far cover a wide range of geographies, which is good, but most focus on variations over time or between regions in a single country. Two studies did find that GI use results vary based on other location characteristics, such as the nature and distribution of GI [31] and COVID-19 lockdown regulations [35]. Casa Nova et al. [29] discussed, but did not test for, an association with population density.

### 4.2. Impacts of GI on Non-COVID-19 Health Outcomes within the Pandemic

The fifteen articles identified in theme 2 provide a much more straightforward interpretation, providing strong evidence of the positive impact of GI use on physical and mental health within the context of the COVID-19 pandemic and associated social distancing measures. Though the pandemic and subsequent control strategies restricting movement had an overall negative impact on mental and physical health, the studies reviewed here consistently found that negative impacts with regard to a wide range of physical and mental health indicators were decreased for those who were able to actively use green infrastructure in the form of public parks, private gardens and yards, and other natural areas. These findings were consistent across GI types, particularly with regard to mental health outcomes. As with the literature on COVID-19 outcomes, potential variations in impacts across demographic groups remain largely unexplored.

Though these studies overall presented a more unified set of findings and were all analyzed at the individual level, several do have some methodological limitations that prevent them from definitively proving causation. All but three of the studies involve population surveys and compare respondent health across levels of GI use. With this approach, findings that GI use are associated with better health can mean that the GI use has improved health, but these results could also represent a bias whereby those with poorer mental and physical health were less interested in using GI as a result of their health status. Three of the studies were able to more directly measure the impact of GI use by measuring individual outcomes before and after GI use [37,41,47], though they lacked controls to represent that effect of not using GI. While the high level of agreement across studies offers support for their conclusions on the benefits of GI use, studies with a random assignment of participants to control and use categories would be even stronger.

### 4.3. Implications

The findings detailed here suggest that green infrastructure such as parks and yards has played a role in shaping the progression and impacts of the COVID-19 pandemic. Though spending time in parks necessitated being out in public and was associated in some areas with increased spread of COVID-19, it nevertheless had a smaller impact than other out-of-home activities, and was demonstrated to be protective to the extent that it replaced riskier activities. Green infrastructure use also helped to mitigate the negative health impacts of COVID containment measures. Given these potential benefits of GI use, it is additionally important to consider the extent to which people were able to access GI during the pandemic, particularly during lockdown restrictions.

The COVID-19 pandemic significantly altered people’s behavior and mobility patterns as people minimized movement in an attempt to curtail the spread of the virus. One potential implication of this is the importance of *local* green infrastructure to enable use. Overall measures of mobility and travel decreased significantly during quarantine and lockdown phases of the pandemic [51,52], and studies looking at park or GI use specifically have produced mixed results, with some people and areas seeing significant increases in GI use and others seeing decreases. Many of the largest drops in GI use have been observed in more destination-oriented GI, such as national parks [53] and historical gardens [54]. Some studies indicated a shift in GI users towards local visitors and who had not previously visited or engaged in outdoor recreation, suggesting that proximity was a major factor in their visitation decisions [55,56]. Other studies found that even when people maintained or decreased their use of GI, they shifted their use patterns to more local venues (such as birdwatching and use of lawns for children’s play). Prior to the pandemic, distance was an important predictor of green space use [57], and pandemic-related mobility decreases may well have narrowed the potential travel distances to GI even further. Local green infrastructure was also considered to be safer in terms of COVID-19 transmission, as utilizing local green spaces does not necessitate travel and increased interaction with others. If travel is necessary, moving within GI appears to be less risky than other forms of transportation. As the pandemic continues into its second year and new variants increase the likelihood of lockdown, public health officials should consider ways to enable GI use even if other forms of mobility become restricted.

If local green infrastructure became more important during the pandemic as overall mobility decreased, then inequities in the availability of local GI also must be recognized as more significant. Numerous previous studies have shown that poor and minority residents in the US tend to have access to fewer, smaller, and lower-quality green spaces [58,59] and to live in areas with less vegetation [60,61], as well as being treated unequally when they do use GI [62], suggesting they would be less likely to experience the benefits highlighted by theme 2 studies in particular.

For urban planners, the implications are particularly clear. Planners must consider the potential for future pandemics and work towards assuring access to quality GI in close proximity to all. The COVID-19 pandemic has been deeply unequal in its impacts [63,64], and while GI cannot reasonably address all components of that inequality, it can contribute to doing so. In fact, one study of racial disparities in COVID-19 outcomes in the US found that counties with more GI demonstrated lower COVID-19 racial disparities than those with less GI [65]. Given the particular importance of proximity for accessing GI during pandemic conditions, smaller, more dispersed GI may be more beneficial than larger, centralized GI for tackling these inequalities, especially for communities where private GI is limited.

Given the mass shifts in work and mobility experienced during the pandemic, some of which are expected to continue even after the pandemic ends, we have an opportunity to return to a “new normal” that does not necessarily match the way of life of pre-pandemic times. This new normal could be one in which green infrastructure is more intentionally planned and prioritized towards the development of a more equitable and sustainable future in light of climate change and future pandemics. Thus, the COVID-19 pandemic can be seen as an opportunity to regroup and reprioritize for the future, with a potential focus on re-building society to take better and more equitable advantage of the benefits of GI for managing future pandemics.

## 5. Conclusions

The COVID-19 pandemic represents an unprecedented moment for studying the deep integration of green infrastructure and public health. During lockdown, local GI truly played a role as critical infrastructure, protecting mental and physical health for surrounding residents despite significant operational constraints. Though mobility overall increased COVID-19-specific health risks, mobility to parks may be considerably less risky than other destinations. The COVID-19 pandemic has highlighted the potential value of green infrastructure access and its importance in planning for resiliency in the face of inevitable future pandemics. The increased attention to and importance of green infrastructure during COVID-19 has foregrounded equity as a concern for future planning to ensure that safe and high-quality GI is available for all. While much remains to be learned as the pandemic continues to unfold, the importance of green infrastructure for health promotion is clear.

## Figures and Tables

**Table 1:** Summary chart of articles identified in theme 1: the impacts of GI use on COVID-19-specific health outcomes.

Author (Year)	Unit of Analysis		Health Outcomes			Impact of GI Use		
	State/Region/District	Country	COVID-19 Cases	COVID-19 Reproductive Rate	COVID-19 Deaths	Reduced Negative Outcomes	No Impact	Increased Negative Outcomes
Casa Nova et al. (2021) [29]	x		x					x
DePhillipo et al. (2021) [30]	x		x				x	
Johnson et al. (2021) [31]	x		x			x		
Kartal, Depren, and Depren (2021) [32]		x	x		x		x	x
Noland (2021) [33]	x			x				x
Praharaj and Han (2021) [34]	x		x				x	
Tyrovolas et al. (2021) [35]		x	x		x	x		x

**Table 2:** Summary of articles identified in theme 2: the impacts of GI use on non-COVID health outcomes.

Author (Year)	Form of GI Use			Health Outcomes																	Impact of GI Use		
	Self-reported park/green space visits	Self-reported private GI use	Use of unspecified green or natural area	Anxiety	Cancer symptoms	Depression	Fatigue	Life satisfaction	Loneliness	Mental distress	Mental wellbeing	Physical health	Psychological distress	Psychopathology (internalizing)	Psychopathology (externalizing)	Self-esteem	Sleep quality	Somatization	Stress	Subjective wellbeing	Reduced negative outcomes	Increased positive outcomes	No impact
Corley et al. (2021) [36]		x		x							x	x					x				x		
Gola et al. (2021) [37]			x	x		x															x		
Heo et al. (2021) [38]	x			x		x															x		x
Hubbard et al. (2021) [39]	x												x								x		
Jackson et al. (2021) [40]			x																	x	x		
Lee et al. (2021) [41]	x																		x		x		
Lehberger, Kleih and Sparke (2021) [42]	x	x									x											x	
Longman et al. (2021) [43]			x	x		x	x														x		x
Marques et al. (2021) [44]	x	x								x											x		
Mayen Huerta and Utomo (2021) [45]	x										x											x	
Pearson et al. (2021) [46]	x	x			x																x		
Rajoo et al. (2021) [47]			x	x		x													x		x		
Ribeiro et al. (2021) [48]	x	x											x					x	x		x		
Rosen et al. (2021) [49]			x											x	x								x
Soga et al. (2020) [50]	x			x		x		x	x							x					x	x	

## Data Availability

Not Applicable.

## Appendix A. Details of Included Articles

**Table A1:** Authors, title, and theme for all included articles.

Author (Year)	Title	Theme
Casa Nova et al. (2021) [29]	Are mobility and COVID-19 related? A dynamic analysis for Portuguese districts	Theme 1: COVID-specific Outcomes
DePhillipo et al. (2021) [30]	Mobile phone GPS data and prevalence of COVID-19 infections: Quantifying parameters of social distancing in the U.S.	Theme 1: COVID-specific Outcomes
Johnson et al. (2021) [31]	Associations between COVID-19 transmission rates, park use, and landscape structure	Theme 1: COVID-specific Outcomes
Kartal, Depren, and Depren (2021) [32]	The relationship between mobility and COVID-19 pandemic: Daily evidence from an emerging country by causality analysis	Theme 1: COVID-specific Outcomes
Noland (2021) [33]	Mobility and the effective reproduction rate of COVID-19	Theme 1: COVID-specific Outcomes
Praharaj and Han (2021) [34]	Human mobility impacts on the surging incidence of COVID-19 in India	Theme 1: COVID-specific Outcomes
Tyrovolas et al. (2021) [35]	Estimating the COVID-19 spread through real-time population mobility patterns: Surveillance in low- and middle-income countries	Theme 1: COVID-specific Outcomes
Corley et al. (2021) [36]	Home garden use during COVID-19: Associations with physical and mental wellbeing in older adults	Theme 2: Non-COVID Health Outcomes
Gola et al. (2021) [37]	Influence of Nature at the Time of the Pandemic: An Experience-Based Survey at the Time of SARS-CoV-2 to Demonstrate How Even a Short Break in Nature Can Reduce Stress for Healthcare Staff	Theme 2: Non-COVID Health Outcomes
Heo et al. (2021) [38]	Impact of changed use of greenspace during COVID-19 pandemic on depression and anxiety	Theme 2: Non-COVID Health Outcomes
Hubbard et al. (2021) [39]	Are rurality, area deprivation, access to outside space, and green space associated with mental health during the COVID-19 pandemic? A cross sectional study (charis-e)	Theme 2: Non-COVID Health Outcomes
Jackson et al. (2021) [40]	Outdoor Activity Participation Improves Adolescents’ Mental Health and Well-Being during the COVID-19 Pandemic	Theme 2: Non-COVID Health Outcomes
Lee et al. (2021) [41]	Influence of forest visitors’ perceived restorativeness on social–psychological stress	Theme 2: Non-COVID Health Outcomes
Lehberger, Kleih and Sparke (2021) [42]	Self-reported well-being and the importance of green spaces—A comparison of garden owners and non-garden owners in times of COVID-19	Theme 2: Non-COVID Health Outcomes
Longman et al. (2021) [43]	Time in nature associated with decreased fatigue in UK truck drivers	Theme 2: Non-COVID Health Outcomes
Marques et al. (2021) [44]	Home gardens can be more important than other urban green infrastructure for mental well-being during COVID-19 pandemics	Theme 2: Non-COVID Health Outcomes
Mayen Huerta and Utomo (2021) [45]	Evaluating the association between urban green spaces and subjective well-being in Mexico city during the COVID-19 pandemic	Theme 2: Non-COVID Health Outcomes
Pearson et al. (2021) [46]	Increased use of porch or backyard nature during COVID-19 associated with lower stress and better symptom experience among breast cancer patients	Theme 2: Non-COVID Health Outcomes
Rajoo et al. (2021) [47]	Addressing psychosocial issues caused by the COVID-19 lockdown: Can urban greeneries help?	Theme 2: Non-COVID Health Outcomes
Ribeiro et al. (2021) [48]	Exposure to nature and mental health outcomes during COVID-19 lockdown. A comparison between Portugal and Spain	Theme 2: Non-COVID Health Outcomes
Rosen et al. (2021) [49]	Promoting youth mental health during the COVID-19 pandemic: A longitudinal study	Theme 2: Non-COVID Health Outcomes
Soga et al. (2020) [50]	A room with a green view: the importance of nearby nature for mental health during the COVID-19 pandemic	Theme 2: Non-COVID Health Outcomes

## Appendix B. Additional Details of Theme 1 Studies

**Table A2:** Methodological details for theme 1 studies.

Study	Geographic Extent	Unit of Analysis	Methods	Outcome Variable	Explanatory Variables	Detailed Findings	Proposed Mechanism
Casa Nova et al. (2021) [29]	Portugal	Districts	Detrended cross-correlation analysis	Daily new COVID-19 cases	Mobility indices	Mobility in general is positively correlated with new COVID-19 cases, but with a lag of 7–8 days. Parks show a more consistent and lower correlation compared to other forms of mobility.	Lower impact of park mobility compared to other modes suggested to be result of parks as open spaces with lower capacity for contagion. Note potential interaction with population density. Additionally, suggest that containment measures that closed some types of parks may have led to more congestion in open parks, which might make them more prone to increased transmission.
DePhillipo et al. (2021) [30]	USA	State	Multivariable linear regression	COVID-19 infection rates	Mobility in each of 6 groups as percent change from baseline	General association between increased mobility and increased COVID-19. When mobility broken into sectors, significant results only for retail and recreation and grocery and pharmacy mobility categories.	Parks not specifically discussed.
Johnson et al. (2021) [31]	England	Local government administrative areas	Generalized additive model	Daily COVID-19 case rates	Park use relative to other measures of mobility, population density, population clustering, poor health, population over 70, unemployed percent, green space patchiness, green space availability	Mobility was the most significant predictor of case rates, but park use as share of overall mobility associated with decreased case rates. Park use had more effect when patchiness and amount of green space were low.	The positive impact of park mobility is tied to the extent that time spent in parks replaces time spent in riskier areas. Lower movement is better overall, but parks are suggested as safer option if mobility is required.
Kartal, Depren, and Depren (2021) [32]	Turkey	Country	Toda-Yamamoto causality test	Daily number of patients, daily deaths	Walking and driving mobility (Apple data), destination-based mobility (Google).	Causal relationship identified between park mobility and deaths, but not patients.	General discussion of mobility as tied to transmission, but no park-specific mechanism was discussed.
Noland (2021) [33]	USA	State	Log-linear models	COVID-19 effective reproduction rate	Destination-based mobility metrics	Positive correlation between all mobility categories except residential and reproductive rate	Park-based mobility has the weakest relationship (smallest coefficient) of all mobility categories, which authors suggest indicates less viral spread at parks compared to other destinations
Praharaj and Han (2021) [34]	India	State	Poisson log-linear model	COVID-19 cases	Destination-based mobility metrics	No significant relationship between park mobility and COVID-19 cases	
Tyrovolas et al. (2021) [35]	Global, with regional analyses for Latin America, Africa, the Caribbean	Country	Negative binomial mixed model	Daily new COVID-19 cases	Countries’ preparedness in epidemics (INFORM index), COVID-19 testing policy, COVID-19 type of transmission for each country through time, populations’ real-time mobility patterns, their interaction with each level of government control policy, and the country’s income level	Park mobility interacted with government control measures—it exhibited a negative relationship to daily cases overall, but a positive relationship where control measures were in place. This held at the regional level for Latin America and the Caribbean, but not Africa	Individual behavior, dynamic network, and seasonality may be factors, but no discussion offered specifically for parks
